# Letter to the editor: Comments on ‘Striking long-term beneficial effects of single-dose psilocybin and psychedelic mushroom extract in the SAPAP3 rodent model of OCD-like excessive self-grooming’ by Brownstien et al. (2024)

**DOI:** 10.1038/s41380-025-03005-0

**Published:** 2025-04-06

**Authors:** James J. Gattuso, Carey Wilson, Anthony J. Hannan, Thibault Renoir

**Affiliations:** 1 Florey Institute of Neuroscience and Mental Health, Melbourne Brain Centre, University of Melbourne, Parkville, Australia; 2 Faculty of Medicine, Dentistry and Health Sciences, University of Melbourne, Parkville, Australia

**Keywords:** Neuroscience, Drug discovery

Dear Editor,

The growing interest in psychedelic compounds for psychiatric disorders highlights the importance of well-designed preclinical studies [1, 2]. In this context, Brownstein et al. [3] recently reported evidence of potential therapeutic benefits of psychedelic mushroom extract (PME) in the SAPAP3 knockout (KO) mouse model of obsessive-compulsive disorder (OCD)-like behaviours.

A strength of the study is the comparison of psilocybin to psychedelic mushroom extract (PME). This is because psilocybin mushrooms also contain a host of biologically active compounds (such as baeocystin and norbaeocystin), which may produce an entourage effect. An entourage effect refers to the phenomenon whereby the combined effect of various compounds in a natural substance, like psilocybin mushrooms, produces a different (often more potent or beneficial) outcome than any of the individual compounds would produce on their own (e.g., psilocybin) [4].

Additionally, Brownstien et al. [3] used multiple assays of compulsive-like behaviour, such as grooming and the marble-burying test, and anxiety-like behaviour, such as the open-field test, the elevated-plus maze and head-body twitches (which represent tic-like behaviour in SAPAP3 KO mice) [5]. The authors found that a single dose of psilocybin (4.4 mg/kg) and PME led to enduring reductions in grooming behaviour in SAPAP3 KO mice (up to 7 weeks for ‘responsive’ mice and up to 3 weeks for vehicle-treated and ‘non-responsive’ mice). Furthermore, psilocybin and PME reduced anxiety-like and tic-like behaviour, with PME having more of a pronounced effect. Finally, we commend the research team on investigating grooming behaviour well after the single psilocybin and PME administration (i.e., 7 weeks post-treatment) as previous research has found therapeutic effects of psilocybin lasting up to 41 days [6].

Despite the interesting and novel aspects of this study by Brownstien et al. [3], we contend that there are certain methodological approaches, statistical analyses and interpretations in the published work that require further critical discussion.

## Absence of wild-type control mice receiving psilocybin administration

A fundamental concern in the design of this study by Brownstien et al. [3] is the absence of wild-type (WT) control mice in the experiments assessing the behavioural effects of psilocybin and PME. While the study focuses on SAPAP3 KO mice as a model for OCD-like behaviours, the lack of WT controls severely limits the ability to draw robust conclusions about the specificity of psilocybin’s effects in alleviating these behaviours. Without a WT comparison, it is unclear whether the observed effects in SAPAP3 KO mice are unique to this pathological model or whether similar behavioural changes might be seen in healthy, wild-type mice.

In similar preclinical studies, the inclusion of both WT and KO mice is standard practice to help differentiate between treatment effects that are specific to the disorder model versus general effects of the compound [7–10]. This omission leaves a critical gap in the interpretation of the data, particularly concerning whether psilocybin and PME ameliorate excessive grooming and anxiety specifically in the SAPAP3 KO model, or if they have more generalised effects which extend to WT mice. Additionally, considering the findings that psilocybin has previously reduced marble burying in WT NMRI mice [11] and WT ICR mice [12] it is possible that psilocybin could also reduce grooming in WT animals but to a lesser extent than SAPAP3 KO animals. Having WT control groups would allow a comprehensive statistical analysis looking at potential interaction between genotype and treatment. Indeed, while both WT and SAPAP3 KO mice could respond to treatment, they could do so in a distinct/different fashion. For instance, if psilocybin and PME significantly reduced grooming behaviour in WT animals in conjunction with SAPAP3 KO animals, it could mean that the effects of psilocybin and PME are not purely anti-compulsive (as WT mice do not compulsively groom, but rather grooming is a natural hygienic behaviour that is sensitive to stress). Therefore, the effect could be more generalised and related to altering the stress response, or sensory and tactile perception—for instance, psilocybin could alter sensory processing and attention, potentially reducing grooming by shifting focus away from bodily sensations or environmental stimuli that might trigger grooming.

We note that the lack of WT drug-treated controls in Brownstien et al. [3] was not due to breeding issues, as both WT and SAPAP3 KO mice were derived from heterozygote x heterozygote breeding mice. We are therefore unclear as to the reason WT mice were not used to assess treatment effects, and we also note that this limitation was not addressed in the discussion.

## Lack of exploration of sex differences

Despite the importance of sex as a biological variable [13, 14], Brownstien et al. [3] did not fully explore potential differences in the behavioural response to psilocybin and psychedelic mushroom extract between male and female mice. Given the well-documented sex-specific responses to serotonergic psychedelics [1, 14], we suggest that this may represent an important gap in the current analytical approach.

For instance, Tylš et al. [15] found that psilocin induced greater reductions in locomotion and pre-pulse inhibition in male rather than female rats. However, the female rats displayed a greater number of wet-body shakes (a proxy of the hallucinogenic experience) compared to males. These results align with the findings that the synthetic serotonergic psychedelic 2,5-Dimethoxy-4-iodoamphetamine (DOI) induced a greater number of head-twitches in female mice compared to male mice [16]. In alignment with these findings, Meinhardt et al. [17] also found more pronounced hypolocomotor effects of psilocybin in male rats. Finally, we recently published a paper reporting sex-dependent behavioural responses in grooming behaviour following psilocybin administration [9]. We found that psilocybin (1 mg/kg, i.p.) reduced grooming behaviour in male SAPAP3 KO mice 3 and 8 days after injection. However, in female mice there was an overall treatment effect, thus, psilocybin reduced grooming in both female WT and SAPAP3 KO across all time points, suggesting sex-specific effects of psilocybin on grooming behaviour [9]. Additionally, prior work has found that young-adult female SAPAP3 KO mice spontaneously groom less compared to male KO mice, which could explain why female KO mice are less sensitive to psilocybin’s effect on grooming behaviour, as there is already a lower level of grooming in basal conditions [18]. Finally, on the cellular level, psilocybin has been found to increase dendritic spine density in the medial prefrontal cortex of C57BL/6J mice to a greater extent in female rather than male mice [19].

Although the authors assessed behaviours in male and female mice and report their results in the supplementary file, they did not provide the male and female data for the marble-burying test, open-field test or grooming at 2- and 12-days post treatment. The authors briefly mentioned “There was no effect of treatments on the grooming phenotypes when comparing male and female mice” (page 7) (Brownstien et al., 2024) [3]. However, the authors did not report on any statistical testing to analyse whether psilocybin differentially affected male and female SAPAP3 KO animals (by running comprehensive ANOVAs analysing the variance due to 3 factors, namely treatment, days and sex). We managed to analyse the supplementary findings and found no significant differences between sexes (*p* = 0.427) or sex x treatment interaction (*p* = 0.785) on grooming behaviour 21 days after treatment.

Given the existing literature on sex-specific responses to psychedelics and serotonin-mediated behaviour [14], we believe further exploration of sex differences would not only enrich the study but could also provide more tailored therapeutic insights. Additionally, in light of the previous literature suggesting sex-dependent response in psilocybin-induced behavioural outcomes, it would have been appropriate for the authors to discuss their lack of sex differences in the context of the broader literature, and we encourage this for future publications.

## Interpretation of behavioural findings

The central finding of long-term benefits of a single dose of psilocybin or PME in SAPAP3 KO mice in Brownstien et al. [3] is compelling, and we are pleased to have recently observed similar results in this mouse model [9]. However, Brownstien et al. [3] reported a ‘striking’ increase in grooming behaviour over time in the vehicle-treated SAPAP3 KO group (118.71 ± 95.96% increase over 21 days), which drove the statistically significant effect despite there only being a relatively modest reduction in grooming behaviour in KO animals of 14.60 ±  17.90% (21 days post-treatment). These findings are unusual because vehicle-treated SAPAP3 KO mice typically exhibit stable grooming behaviours around 7 days after administration and with repeated testing exposure [8, 9]. It would have been informative for the authors to discuss why there was such a large percentage increase in the vehicle-treated SAPAP3 KO mice over time. For example, if the increase in grooming behaviour in the vehicle-treated SAPAP3 KO mice was due to stress effects (possibly from the other behavioural assays run before grooming was assessed), then psilocybin’s effect may be less about reducing compulsive behaviour, but rather about bolstering against stress and increasing stress resilience. On that note, it would be interesting to assess the behaviour of SAPAP3 KO mice under conditions of repeated stress, indeed there is yet to be a comprehensive characterisation of the stress response of SAPAP3 KO mice, especially whether their OCD-like phenotypes worsen with exposure to chronic stress paradigms.

In addition, the exclusion of lesioned SAPAP3 KO animals and WT controls might make it difficult to elucidate—with precision—the exact drivers of Brownstien et al. (2024)’s findings [3]. For example, it would have been interesting to know whether psilocybin would also reduce grooming in wild-type mice (as we recently described [9]), as this could mean that the reduction in grooming induced by psilocybin may not have been purely via anti-compulsive effects. Relatedly, we note that the presence of lesions indicates that the grooming behaviour is compulsive—because, despite causing self-harm, they cannot stop. Thus, only measuring the effects of psilocybin and PME on non-lesioned animals may mean that their excessive grooming behaviour is not purely compulsive and a reduction in this grooming behaviour may not be exclusively anti-compulsive. A possible alternative would be to include mice that start to develop body lesions; however, upon early inspection of lesions, the mice can be treated by clipping their hind-paw nails which often results in significant improvement in skin health [5]. Interestingly, the authors (if sufficiently powered) could have documented whether lesioned SAPAP3 KO mice behaved and/or responded differently to the drug treatment [20].

## Lack of mechanistic experiments

The focus of both Brownstien et al. [3] and Gattuso et al. [9] was on demonstrating the long-term therapeutic potential of psilocybin in reducing excessive self-grooming and anxiety-like behaviours in SAPAP3 KO mice. However, the inclusion of mechanistic experiments utilising pharmacological blockade with selective 5-HT_1A_, 5-HT_2A_ and 5-HT_2C_ antagonists, could have significantly improved the strength of both studies. Such findings could provide critical evidence to support or refute the hypothesis that these serotonergic receptors are directly involved in the anti-compulsive-like effects of psilocybin and PME. These findings would be crucial in the context of the broader literature, as recent studies have found that psilocybin can reduce rodent marble burying independently of 5-HT_1A_, 5-HT_2A_ and 5-HT_2C_ receptor activation [11, 12].

We propose that these receptor antagonism studies could also help differentiate between the roles of psilocybin and PME in targeting distinct receptor profiles. PME, which contains additional entourage compounds like baeocystin, might engage alternative or supplementary mechanisms compared to synthetic psilocybin alone, which was previously indicated in PME anti-compulsive-like effects [21]. Additionally, these pharmacological insights could guide the development of targeted therapeutic strategies that leverage specific receptor pathways to optimise clinical outcomes in OCD treatment.

## Conclusions

We acknowledge the valuable contributions of Brownstien et al. [3] to the field of psychedelic research and OCD treatment, particularly regarding the effects of psilocybin on compulsive behaviours. Notably, the sustained reduction in excessive grooming behaviour following psilocybin administration in SAPAP3 KO mice, first described in Brownstien et al. [3], has also been found in our own laboratory (Gattuso et al., 2024) [9] —in a study published just a month later—demonstrating robust and complementary findings across both studies despite methodological differences. Additionally, each paper contains unique strengths and weaknesses and when viewed together, they add a more detailed understanding of psilocybin’s anti-compulsive-like effects.

For example, while Brownstien et al. [3] did not incorporate WT psilocybin control mice, analyse sex differences, or include lesioned SAPAP3 KO animals, these aspects were addressed by Gattuso et al. [9], thereby providing additional insights. Conversely, Brownstien et al. employed a broader range of anxiety-related behavioural assays, which may explain the anxiolytic effects observed in their study but not detected by Gattuso et al. [9]. Additionally, Gattuso et al. [9] assessed psilocybin’s behavioural effects up to 8 days post-administration, whereas Brownstien et al. [3] extended the observation period for grooming behaviour to 7 weeks.

A unique strength of Brownstien et al. [3] lies in their comparative analysis of psilocybin and psilocybin mushroom extract (PME), in contrast to Gattuso et al. [9], where we focused exclusively on psilocybin. Furthermore, in Gattuso et al. [9] we provided novel insight by correlating acute hallucinogenic-like behaviour (psilocybin-induced head twitches) with reductions in grooming behaviour, suggesting that the head-twitch response may be a critical component of psilocybin’s anti-compulsive effects in male SAPAP3 KO mice. Finally, it must be noted that both studies used different psilocybin doses (1 mg/kg [9] vs 4.4 mg/kg [3]) but the direction of findings was relatively consistent (i.e., psilocybin reduced excessive grooming duration). Together, these studies offer complementary data and perspectives on the therapeutic potential of psilocybin in OCD models.

As we have critically evaluated the results from Brownstien et al. [3], we conclude that addressing the various concerns outlined above would strengthen the overall impact of the study and provide clearer guidance for future clinical applications. We hope that our suggestions are taken in the spirit of constructive scientific discourse, and we look forward to seeing continued advancements in this exciting area of research.
